# Chronic Kidney Disease-Associated Pruritus: A Glance at Novel and Lesser-Known Treatments

**DOI:** 10.7759/cureus.21127

**Published:** 2022-01-11

**Authors:** Sayed Elhag, Nancy Rivas, Sreedevi Tejovath, Nadiah Mustaffa, Nadira Deonarine, Muzaffar Abdullah Hashmi, Sindhura Yerneni, Pousette Hamid

**Affiliations:** 1 Internal Medicine, California Institute of Behavioral Neurosciences & Psychology, Fairfield, USA; 2 Neurology, California Institute of Behavioral Neurosciences & Psychology, Fairfield, USA

**Keywords:** dialysis, hemoperfusion, itching, ckd pruritus, ckd, uremic pruritus

## Abstract

Chronic kidney disease-associated pruritus (CKD-aP), also known as uremic pruritus, has been associated with increased mortality and lower quality of life among patients with chronic kidney disease (CKD). The relentless nature of the condition is mainly due to its diverse and complex etiologies, which are still being studied. Despite the introduction of many agents to treat it, the resolution rates of CKD-aP still remain unsatisfactory. This study sought to review the lesser-known/novel treatments and establish a relationship between their mechanism of action and the proposed etiologies implicated in CKD-aP. We also discuss the role of dialysis modification in managing CKD-aP.

A decent proportion of the reviewed studies have proposed that the agents analyzed in them act through hampering inflammation. Interestingly, the results of two agents alluded to the role of dysbiosis in CKD-aP. The addition of hemoperfusion to the dialysis regimen of patients with CKD-aP improved the severity of their symptoms.

The featured treatments could be tried in patients with intractable symptoms. However, additional research is needed to confirm the findings reported in these studies. A better understanding of the pathologic mechanisms is required to help guide the development of agents that can better treat CKD-aP.

## Introduction and background

Chronic kidney disease-associated pruritus (CKD-aP), sometimes called uremic pruritus, is a common condition affecting patients with CKD. A meta-analysis of several observational studies has reported a prevalence of at least 50% in patients on dialysis [1]. CKD-aP is more than just an irritating itch; in fact, it has far-reaching effects and has been associated with increased morbidity and mortality and decreased quality of life [2-4]. CKD-aP affects patients on hemodialysis and peritoneal dialysis to a similar degree; however, a study on the topic has found that it affected patients on hemodialysis to a lesser extent [1,5]. Interestingly, it has been observed that CKD-aP may persist even after renal transplantation; a study performed in 2020 to determine the prevalence of chronic pruritus among post-renal transplantees revealed a prevalence of 12% [6]. The presentation of CKD-aP is highly variable; it can be localized or generalized and can occur intermittently or persistently [7,8]. Also, the patients usually do not exhibit skin lesions [7].

The condition's pathophysiology is complex and is thought to be mediated by the interplay of several mechanisms that are yet to be fully understood [8]. Determining the etiology is further complicated by the potential presence of specific factors associated with a higher chance of developing CKD-aP, such as having hepatitis C and using arteriovenous graft as vascular access for hemodialysis [9-12]. Raised serum calcium, parathyroid hormone (PTH), and phosphorus have also been associated with CKD-aP. The exact mechanism as to how they bring about pruritus is not well elucidated [11,13]. Dry skin has been implicated in the pathogenesis of CKD-aP through a myriad of processes such as a change in skin pH and increased urea excretion in the skin [13].

**Figure 1:**
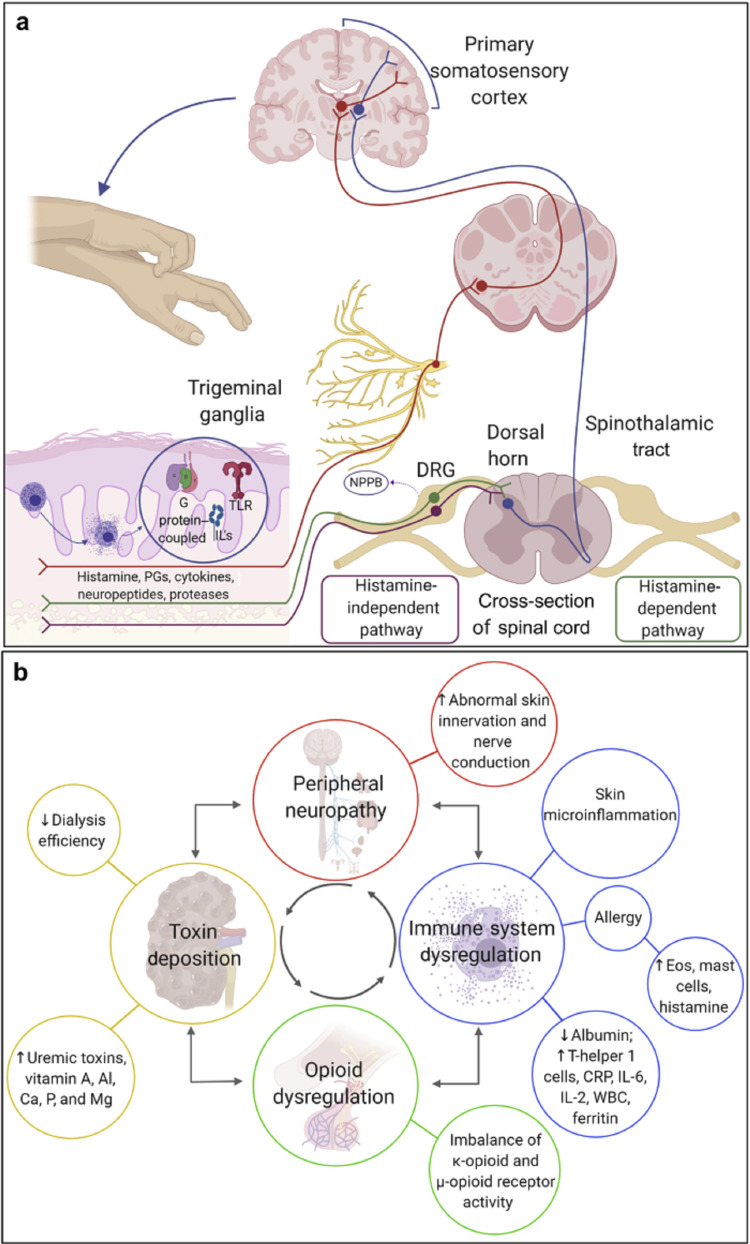
Pathogenesis of CKD-aP CKD-aP: chronic kidney disease-associated pruritus Image courtesy: Verduzco HA, Shirazian S. CKD-associated pruritus: new insights into diagnosis, pathogenesis, and management. Kidney International Reports. 2020 Sep 1;5(9):1387-402 [8]

Additionally, the evidence supporting the role of opioid receptors in the pathogenesis of CKD-aP has been strengthened most recently by the KALM-1 trial, which reported pruritus relief in patients treated with difelikefalin [14]. Naltrexone, however, showed conflicting results that suggested the lack of participation of opioids in the condition's pathophysiology [15-17]. Moreover, the contribution of immune dysregulation has been supported by the finding of raised inflammatory markers such as C-reactive protein (CRP) in patients with CKD-aP and their positive response to immunomodulatory drugs [8,13,18]. Furthermore, gabapentin has been shown to be beneficial in treating CKD-aP; this observation suggests a role of neuropathic dysregulation in the pathogenesis of CKD-aP [8,19]. The role of neuropathic dysregulation is further backed by the finding of raised serum levels of neurotrophin-4 and brain-derived nerve growth factors in uremic pruritus patients [20]. Lastly, certain uremic toxins and pruritogenic substances have been implicated in the pathogenesis of CKD-aP. A study conducted by Wu et al. found 22 metabolites that were substantially different between patients with severe uremic pruritus and those with mild uremic pruritus [21]. Interestingly, elevated serum aluminum was associated with pruritus, and its level correlated with the intensity of the itch, but this was not observed in the DOPPS II trial [11,22].

Kt/v, a widely used measure of hemodialysis adequacy, has an inconsistent relationship with pruritus. The DOPPS II study reported no association between Kt/v and pruritus [11]. However, the findings of a cohort study by Ko et al. showed decreased pruritus intensity with a Kt/v target of ≥1.5 [23]. The authors also reported lower pruritus intensity using high-flux dialyzers. The use of polymethylmethacrylate membrane dialyzer, a biocompatible membrane, may relieve pruritus [24].

The treatment of CKD-aP usually comprises various different agents. After obtaining a medical history and examining the patient to exclude similar conditions, it is generally recommended to start the patient on skin moisturizers [25]. However, there are no guidelines concerning the order of treatment agents to be used [26]. Several studies have been performed to address this problem [8,27]. Gabapentin, opioid receptors modulators, antihistamine, topical steroids, and ultraviolet B (UVB) are a few of the agents currently being used [9]. Alternative medicine techniques such as acupuncture have also been used for CKD-aP; a systematic review on the utility of acupuncture and acupressure for uremic pruritus has shown success, but the authors have recommended performing more trials on the topic [28].

A concern regarding the reliability of current medications available for CKD-aP is the inconsistent results in terms of their efficacy and constraints regarding study design [29]. Our incomplete understanding of the complex pathologic processes that bring about CKD-aP is also to blame. Additionally, there are concerns regarding the misuse of gabapentin and the pending adoption of nalfurafine in some countries [25].

This paper aims to review lesser-known treatments for CKD-aP and how their mechanism of action relates to the pathogenesis of CKD-aP. The paper also will look into the contribution of hemodialysis alterations to the treatment of CKD-aP.

## Review

Methodology

The evidence for this paper was collected from articles indexed on PubMed and Google Scholar. The search terms used were "CKD associated pruritus," "Uremic pruritus," "ESRD pruritus," and "Treatment of uremic pruritus". Criteria for article selection were as follows: articles in the English language that were published within the last five years.

Pharmacological treatment

Montelukast 

Mahmudpour et al. conducted a double-blinded trial on the efficacy of montelukast in treating uremic pruritus by utilizing the visual analog scale (VAS) and detailed pruritus score to assess pruritus intensity. The study reported a significant reduction in the pruritus scores in the montelukast group. Additionally, serum CRP among the montelukast group showed a statistically significant reduction compared to the placebo group [30]. Montelukast acts on cysteinyl leukotriene receptors for leukotrienes D4 and E4, thereby inhibiting the downstream inflammatory effects of these leukotrienes; since mast cells contain the leukotriene receptors, it could explain montelukast's antipruritic effect [31].

Dupilumab 

Zhai et al. tested the efficacy of dupilumab on patients with chronic pruritus, including uremic pruritus. The study had five patients with uremic pruritus; they all failed to respond to topical steroids and topical calcineurin inhibitors. They were initiated on a bolus dose of dupilumab (600 mg) and then 300 mg every two weeks. The patients were then followed up for 12 weeks. All patients reported an improvement in the numeric rating scale itch intensity (NRSi). However, it should be noted that only one patient was on hemodialysis [32]. A case report documenting the use of dupilumab in treating a 61-year-old patient with uremic pruritus also reported successful outcomes. Prior to initiating dupilumab, the patient had been on narrow-band UVB phototherapy combined with the use of several agents, including doxepin, pregabalin, and naltrexone at least since the age of 55 years. The patient showed remarkable improvement at the end of six months of treatment [33]. The authors suggest that dupilumab may have relieved the itch by blocking interleukin-4 or interleukin-3 (IL-4 or IL-13) on sensory nerve cells [33]. Oweis et al. have reported IL-13 levels correlating with the severity of uremic pruritus [34]. Given that both studies had patients with refractory pruritus, it is reassuring that dupilumab can be tested in future studies to treat intractable pruritus. However, due to the high cost of dupilumab, it is doubtful that insurance companies will authorize the off-label use of the drug.

Nemolizumab 

A randomized, double-blind, placebo-controlled clinical trial was conducted on the itch-reducing effect of nemolizumab. The study subjects were divided into five groups; three groups received nemolizumab at a dose of 0.125 mg/kg, 0.50 mg/kg, and 2 mg/kg, respectively. The fourth group was administered nalfurafine hydrochloride, and the fifth group received a placebo. The study observed no statistically significant improvement in pruritus severity (based on the VAS) between nemolizumab and the placebo group. Also, there was no link between baseline serum IL-31 levels and pruritus score (VAS) in the 48 individuals who received trial therapy. Patients with IL-31 levels of at least 0.86 pg/mL who received nemolizumab demonstrated a decrease in VAS after treatment compared to patients with IL-31 levels <0.86 pg/mL. Neither the placebo nor the nalfurafine groups showed this trend. Surprisingly, the response in the placebo group was higher than that in the nalfurafine group. Nemolizumab is a monoclonal antibody that targets IL-31 [35]. Elevated IL-31 has been found in patients with uremic pruritus [34]. The study findings suggest that nemolizumab is beneficial in a subset of uremic pruritus patients with a high IL-31. Also, the observation that baseline IL-31 did not correlate with baseline VAS score indicates that IL-31 does not correlate with pruritus severity.

Cannabis 

A study mentioned in a review by Rein et al. reported an 81% improvement in pruritus among 21 hemodialysis patients after three weeks on a topical agent containing two endocannabinoids [36,37]. The mechanisms by which cannabis is believed to ameliorate the itch are multifold: stimulation of the cannabinoid receptor 1 (CB1) in the central nervous system, action on the transient receptor potential cation channel subfamily V-1 (TRPV-1) present on sensory neurons, modulation of immune cells through cannabinoid receptor 2 (CB2), and reduction in activation of mast cells [38]. Capsaicin acts on TRPV-1, which explains why capsaicin is effective in treating uremic pruritus [9,38]. The study featured in the review employed palmitoylethanolamide (PEA), a ligand for TRPV-1 channels, which has no direct interaction with CB1 and CB2; this suggests that the efficacy and mechanism of action are similar to those of capsaicin. A future study could compare topical PEA with capsaicin to determine any significant difference between the two drugs. 

Sodium Thiosulphate IV 

A retrospective trial studying the therapeutic effect of sodium thiosulphate in hemodialysis patients complaining of uremic pruritus concluded that patients receiving sodium thiosulphate reported significant relief from pruritus compared to the control group. Given that sodium thiosulphate is used to treat calciphylaxis, one might assume that it ameliorates the pruritus through the removal of calcium; however, no change was observed in calcium and phosphorus levels between the groups [39]. The mechanism of action seems to be non-specific, and further research is needed to identify the mechanism of action.

Butyric Acid Derivative

An experimental study conducted on skin *Cutibacterium acnes (C. acnes)* and a butyric acid derivative about their role in uremic pruritus reported that the number of *C. acnes* was lower on the skin of CKD patients with pruritus compared to CKD patients with no itch. Secondly, a butyric acid derivative, N-(2-(2-Butyrylamino-ethoxy)-ethyl)-butyramide (BA-NH-NH-BA), topically applied on mice injected with calcium phosphate was found to have the most significant effect on the reduction of pruritus and the reduction in the upregulation of IL-6 [and ultimately extracellular signal-regulated kinases 1/2 (ERK 1/2)] than *C. acnes* with glucose. The authors observed that the solubilizing ability of butyric acid is less efficient when compared to propionic acid and acetic acid; however, butyric acid was able to diminish IL-6 upregulation by the deposited calcium phosphate in the skin. Interestingly, the authors also observed no change in ERK 1/2 in IL-6 knockout mice, which confirmed the essential role of IL-6 in the upregulation of ERK 1/2. 

Calcium phosphate accumulation in the skin leads to the expression of IL-6, which in turn activates ERK 1/2 in the dorsal root ganglia leading to increased sensitivity to certain pruritogens [40]. Butyric acid derivatives obtained by fermentation of glucose by *C. acnes* dissolve the calcium phosphate and help relieve the itch [40]. This research suggests that calcium phosphate deposition in the skin is an etiological factor in uremic pruritus. Moreover, the role of inflammation through IL-6 upregulation is believed to mediate the itch through the activation of ERK. However, human studies need to be performed to confirm these findings in research and assess safety.

**Figure 2:**
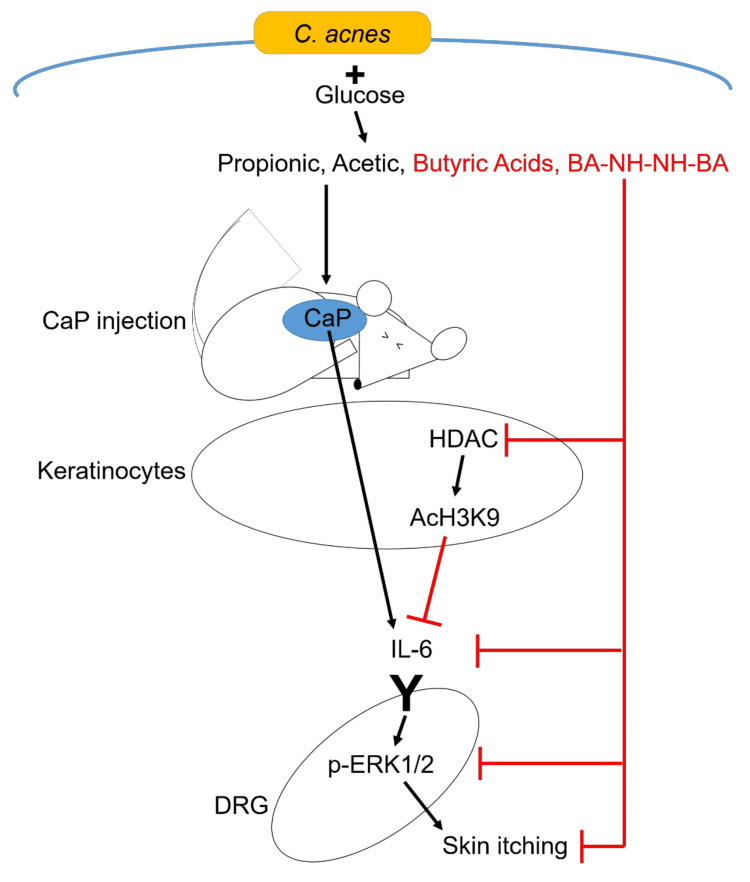
Antipruritic effect of butyric acid and mechanism of calcium phosphate in pruritus Image courtesy: Keshari S, Wang Y, Herr DR, et al. Skin cutibacterium acnes mediates fermentation to suppress the calcium phosphate-induced itching: a butyric acid derivative with potential for uremic pruritus. Journal of Clinical Medicine. 2020 Feb;9(2):312 [40]

Non-pharmacological treatment 

Omega-3 Fatty Acids 

A randomized controlled crossover trial tested the role of omega-3 fatty acids in the treatment of CKD-aP among patients with continuous ambulatory peritoneal dialysis (CAPD); the study assessed the severity of itch intensity using VAS. The study reported significant improvement in the VAS score in both intervention periods [41]. The results of this study are comparable with those of another randomized controlled crossover study [42]. Several anti-inflammatory mechanisms were theorized to have caused the change, such as the reduced activity of the lipoxygenase pathway [43].

Baby Oil 

Singh et al. conducted a study on the effectiveness of baby oil in treating uremic pruritus among patients on hemodialysis. The study subjects were divided into an intervention and control group. The pruritus was assessed using a numerical rating system, and the post-intervention assessment was obtained after 10 days. The study reported a statistically significant improvement in pruritus intensity in the intervention group. Besides the emollient properties of baby oil, the product used in the study contained vitamin E, which aids with pruritus [44,45].

Violet Oil 

Khorsand et al. ran a randomized control trial on the role of violet oil with or without massage on the severity of pruritus in hemodialysis patients. The study population was divided into two groups; one group used topical violet oil, and the other used topical violet oil and massage. The two groups reported improved dryness scores. However, only the group that used topical violet oil with massage reported statistically significant results regarding pruritus score (VAS). The antipruritic effects of the violet plant can be attributed to sitosterol, a substance similar to analogs of hydrocortisone, which confers anti-inflammatory effects when applied to the skin. Recent studies have shown the presence of tiny nerve endings on the skin that transmit itch sensation, and these fibers are similar to those carrying pain sensation (type C fibers) [46,47]. Massage releases endorphins that might act on these nerve endings, thus alleviating the pruritus [48].

Ostrich Oil 

A clinical trial conducted on the therapeutic effect of ostrich oil in reducing the severity of pruritus and improving quality of life in hemodialysis patients reported statistically significant results in those regards. Patients were divided into two groups and were asked to use the intervention for one month. There were no observed changes in the pruritus score during the first and second week; however, during the last two weeks of the trial, there was a reduction in the pruritus score in the group applying the ostrich oil. Oleic acid and linoleic acid are some of the ingredients of ostrich oil, and they relieve pruritus by providing moisture to the skin. Also, linoleic acid is metabolized to an anti-inflammatory prostaglandin analog [49]. The absence of significant changes in pruritus during the first two weeks may discourage physicians from recommending this agent.

Melatonin

A randomized controlled crossover trial conducted by Baharvand et al. studied the antipruritic effect of melatonin on patients with uremic pruritus. The study subjects took either melatonin or placebo for two periods of two weeks, separated by a washout period of one week, and the patients were assessed using the VAS and the 12-item pruritus severity scale questionnaire. The carryover effect was not statistically significant. The study reported better relief from pruritus with melatonin when compared to placebo. It also reported better sleep quality with melatonin [50]. Melatonin production is thought to be decreased in CKD due to the reduced activity of serotonin N-acetyltransferase synthesis [51]. Melatonin is an immunomodulator, and when supplemented, it is thought to relieve the pruritus through an anti-inflammatory mechanism similar to the way it improves the pruritus in atopic dermatitis [50,52].

Charcoal 

Cupisti et al. reviewed studies on the role of charcoal in treating pruritus in CKD caused by pruritogenic uremic toxins. The authors concluded that charcoal could be suggested to patients with severe refractory pruritus but recommended more research due to conflicting evidence. Also, oral charcoal can affect the absorption of certain drugs given to patients, which has not been studied by many trials [53]. Charcoal seems to relieve pruritus by absorbing small molecules responsible for inducing the itch, such as indoxyl sulfate and p-Cresyl sulfate; these substances have been found to amplify protease-activated receptor-2 expression on the skin, which is linked to non-histaminergic itching [54]. The study also highlights the role of dysbiosis in CKD and its possible contribution to pruritus.

Vegetarian Diet 

A cross-sectional study was conducted to find the correlation between a vegetarian diet and uremic pruritus. Pruritus was assessed using the VAS and pruritus scale. Pruritus and inflammatory markers were found to be lower in the vegetarian group (n=15); although the patients were adjusted for dialysis frequency and modality, the low number of participants might have affected the observed finding [55]. Interestingly, six patients in the non-vegetarian group switched to a vegetarian diet for two months, and their CRP and IL-2 decreased significantly. The study results suggest that a vegetarian diet seems to attenuate the inflammatory process mediating the pruritus, which could be due to decreased T cell activity, given that IL-2 is a product of its activation. It would have been interesting if p-Cresyl sulfate and indoxyl-sulfate had been tested to corroborate their relationship with the diet [53].

**Figure 3:**
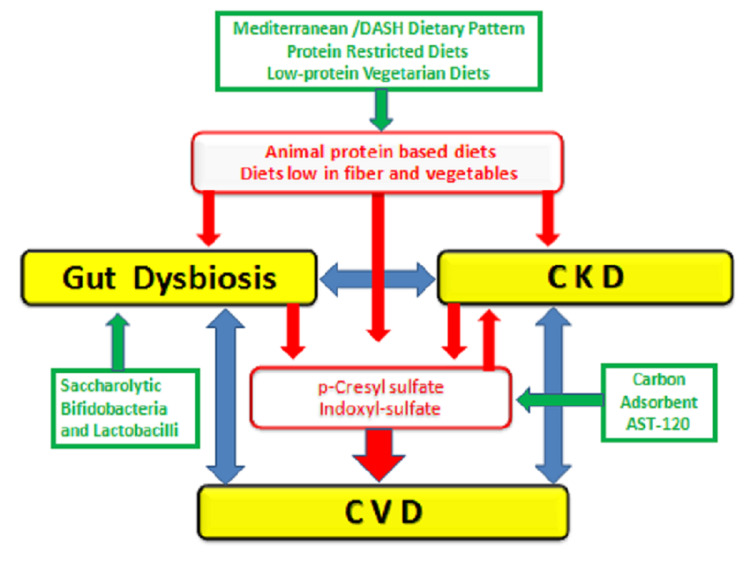
Relationship between chronic kidney disease (CKD), cardiovascular disease (CVD), and microbiota-derived uremic toxins Image courtesy: Cupisti A, Piccoli GB, Gallieni M. Charcoal for the management of pruritus and uremic toxins in patients with chronic kidney disease. Current Opinion in Nephrology and Hypertension. 2020 Jan 1;29(1):71-9 [53]

Dialysis alteration 

Increasing Blood Flow 

A study by Aliasgharpour et al. tested the role of raising the blood flow in patients with uremic pruritus on hemodialysis. The study population was divided into a control group and an experimental group. Two sessions before the intervention, the mean pump velocity was examined and was raised by 25 rounds per minute (rpm) in the first two weeks and 50 rpm in the final two weeks. The frequency of pruritus in the experimental group showed a significant difference after four weeks, while the severity of pruritus between two hemodialysis sessions showed earlier improvement at two weeks [56]. The study participants were matched on age and gender, but the differences in hemoglobin, calcium, CRP, and other biochemical markers were not mentioned. Increasing the blood flow has been associated with better adequacy of dialysis and higher Kt/v [57]. Hence, the results observed by the authors can be attributed to improved dialysis adequacy.

Altering Membrane Flux 

Lim et al. studied the quality of life outcomes (including pruritus) among hemodialysis patients on medium cut-off flux dialyzer vs. high-flux dialyzer. After 12 weeks, the medium cut-off dialyzer group's morning pruritus dispersion was found to be lower. In addition, the medium cut-off dialyzer group had fewer sleep disruptions due to pruritus-related itching. Contrarily, the VAS score in the medium cut-off dialyzer group was higher but not statistically significant compared to the other group (Table 1) [58].

**Table 1:** Comparision in values between baseline and after 12 weeks of intervention in the study by Lim et al.* *[58] MCO: medium cut-off; SD: standard deviation; VAS: visual analog scale

Variables	Baseline			12 weeks		
	MCO (n=24), mean ± SD	High-flux (n=25)	P-value	MCO (n=24)	High-flux (n=25)	P-value
Severity						
Morning	1.92 ± 1.06	1.40 ± 0.50	0.033	1.54 ± 0.72	1.64 ± 0.86	0.667
Afternoon	2.00 ± 1.14	1.72 ± 0.84	0.332	1.88 ± 0.95	1.84 ± 1.07	0.904
Distribution						
Morning	1.42 ± 0.58	1.48 ± 0.71	0.736	1.29 ± 0.46	1.64 ± 0.64	0.034
Afternoon	1.46 ± 0.59	1.56 ± 0.96	0.659	1.38 ± 0.65	1.56 ± 0.71	0.347
Sleep disturbance						
Frequency of waking from sleep	0.83 ± 1.05	0.68 ± 1.28	0.650	0.75 ± 0.85	1.32 ± 1.60	0.126
Frequency of scratching during sleep	0.38 ± 0.92	0.24 ± 0.72	0.571	0.25 ± 0.53	1.00 ± 1.47	0.023
Total score by measuring system	8.58 ± 7.74	7.20 ± 7.58	0.530	6.92 ± 5.98	9.92 ± 8.23	0.152
VAS scoring system						
Morning	2.58 ± 2.24	2.14 ± 2.28	0.496	2.50 ± 1.93	3.34 ± 2.82	0.232
Afternoon	3.04 ± 2.57	2.74 ± 2.53	0.680	3.46 ± 2.32	4.24 ± 3.18	0.333
Average	2.81 ± 2.19	2.44 ± 2.31	0.565	2.98 ± 1.98	3.79 ± 2.91	0.262

Hemoperfusion 

Gu et al. conducted a two-year study on patients on hemodialysis to assess if hemoperfusion combined with hemodialysis improves sleep and mortality rates. The study subjects were divided into groups. One group included 80 patients on hemodialysis only, whereas the other group included 78 patients on hemodialysis combined with hemoperfusion. Hemoperfusion was performed one to two times biweekly for two hours. Besides meeting the primary outcome, patients in the combined hemodialysis and hemoperfusion group demonstrated better amelioration of pruritus and better sleep quality. Additionally, biochemical parameters such as calcium and phosphorus showed improvement [59]. The study findings are reassuring and provide a means of symptom control for those who dislike taking medications or are concerned about polypharmacy.

A trial by Zhang et al. studied patients on combined hemodialysis and hemoperfusion vs. combined hemodiafiltration and hemoperfusion. The authors conducted the trial among a study population of 40 patients. Both groups reported significant improvement in pruritus score and biochemical parameters such as calcium, phosphate, PTH, and β2-microglobulin; however, the combined hemodiafiltration and hemoperfusion group showed much more remarkable improvement [60].

Limitations of the study

This study has some limitations. Most of the selected studies did not have large sample sizes. Additionally, the type of vascular access was not delineated in most studies. Also, only a few studies involved patients on peritoneal dialysis. Lastly, not all agents were included in the study due to time constraints.

## Conclusions

CKD-aP is a condition with diverse etiologies and treatment options. Immune dysregulation and systemic inflammation seem to be the central pathology by which a sizable proportion of the featured agents work. Also, dysbiosis seems to contribute to CKD-aP through an inflammatory mechanism. A study looking into the use of probiotics in CKD-aP would be interesting. 

Additional research is needed to validate the observed effects of the treatments, preferably with a more extensive study population. Future research should also involve patients on peritoneal dialysis. Due to the numerous factors that need to be adjusted for, a single-center study will not be sufficient. Hence, sample collection from multiple centers is recommended. In the meantime, we recommend some of the featured agents as a complementary treatment to patients not responding adequately to their current management. The addition of hemoperfusion or hemodiafiltration is worthwhile and is recommended in CKD-aP patients.
